# Transcriptomic analysis of the upper lip and primary palate development in mice

**DOI:** 10.3389/fgene.2022.1039850

**Published:** 2023-01-06

**Authors:** Sini Cai, Nuo Si, Yanyang Wang, Ningbei Yin

**Affiliations:** ^1^ The Department of Cleft Lip and Palate of Plastic Surgery Hospital, Chinese Academy of Medical Sciences and Peking Union Medical College, Beijing, China; ^2^ Research Center of Plastic Surgery Hospital, Chinese Academy of Medical Sciences and Peking Union Medical College, Beijing, China

**Keywords:** cleft lip with or without cleft palate, RNA sequencing, gene expression profile, development, upper lip and primary palate

## Abstract

**Background:** Normal fusion of the upper lip and primary palate is a complex process involving a series of characteristic and orderly regulated cellular events. Cleft lip with or without palate (CL/P), one of the most common congenital malformations, may be induced by abnormalities in any of these events. However, less is known about the precise regulatory process in the fusion of the upper lip and primary palate.

**Methods:** Lambdoidal junction tissues of mice from embryonic days 10.5, 11.5, and 12.5— three key fusion stages—were acquired for RNA sequencing.

**Results:** Gene expression profiles in distinct fusion stages of mice were identified. Some of the differentially expressed genes (DEGs) have been reported to affect upper lip and primary palate development. However, other DEGs, such as *Krt5*, *Pax1*, *Ambn*, *Hey2*, and *Tnmd*, have not previously been investigated. Gene set enrichment analysis (GSEA) of these DEGs revealed the sequential intensification of Wnt, PI3K-Akt, MAPK, Hippo, and TGF-beta signaling pathways and identified relatively highly expressed genes including *Tnn, Wnt3a*, and *Wnt16*. We also observed substantial alternative splicing events during the fusion of the upper lip and primary palate and identified potentially important genes including *Gtpbp8, Armcx1, Tle3*, and *Numa1*. Protein-protein interaction (PPI) network analysis identified a series of hub genes, including *Col1a2, Fos, Bmp2, Shh, Col1a1, Wnt3a, Anxa1, Gem*, etc.

**Conclusion:** Overall, the results of this study provided a comprehensive analysis of the development of the upper lip and primary palate. Our work provides insight into future studies of normal upper lip and primary palate development and the etiology of CL/P.

## 1 Introduction

Cleft lip with or without palate (CL/P) is one of the most frequent malformations with complex etiology. This condition affects the upper lips and oral cavity, with individual effects on speech, appearance, and psychology, which ultimately impacts human health and well-being (Mossey et al., 2009; Dixon et al., 2011). The estimated morbidity worldwide is 1.7/1,000, with considerable ethnic and geographical variation (Mossey et al., 2009). While previous studies showed that both genetic and environmental factors play important roles in CL/P pathogenesis, the detailed mechanisms remain unelucidated (Mossey and Little, 2009). Since CL/P occurs in the early stage of embryonic development and its recurrence is common and unpredictable, the healthcare burden is substantial (Dixon et al., 2011). Thus, there is an urgent need to identify the characteristic molecular alterations during the embryonic development of the upper lip and palate and provide potential targets for clinical practice.

The mouse craniofacial development process is remarkably like that of humans; thus, most studies select mice for the construction of CL/P models. The mammalian upper face is formed by the ordered fusion of the three bilaterally paired identifiable primordial structures around the stomodeum, namely, the maxillary prominence (MxP), the lateral nasal (LNP), and the medial nasal (MNP) (Neskey et al., 2009; Li et al., 2019). The upper lip, palate, and alveolar ridge are formed during these fusion processes (Graf et al., 2016). The fusion of the upper lip and primary palate in mice begins around embryonic day 10.5 (E10.5) and finishes by E12.5 (Li et al., 2019). Several signaling pathways such as Wnt, Fgf, Shh, and BMP signaling pathways reportedly affect the fusion processes; however, the detailed mechanisms remain unknown (Meulemans and Bronner-Fraser, 2004; Patthey et al., 2009; Stuhlmiller and García-Castro, 2012).

High-throughput sequencing has been widely applied to study the characteristic molecular alterations among distinct stages of development (Reuter et al., 2015). Several microarray analyses have discovered candidate pathogenic gene/loci during murine palatogenesis (Schoen et al., 2017); however, no complete analyses of gene expression patterns during the normal fusion of the upper lip and primary palate have been reported. Especially, the involvement of alternative splicing events has been demonstrated in normal tissue and organic development (Baralle and Giudice, 2017). Missense, splice site, and regulatory region variants in interferon regulatory factor 6 (IRF6) reportedly cause CL/P (Sylvester et al., 2020). The ablation of epithelial splicing regulatory protein (Esrp1) in mice also led to CL/P (Bebee et al., 2015). Herein, we present data on differentially expressed genes and analyze the signaling pathways, signal-gene networks, alternative splicing events, and protein-protein interactions at key stages in upper lip and primary palate development by RNA sequencing (RNA-Seq). We identified and validated stage-specific genes and pathways. Our results regarding upper lip and primary palate development suggested deeper characteristic molecular mechanisms to investigate in future studies, which could contribute to a better understanding of CL/P pathogenesis.

## 2 Materials and methods

### 2.1 Samples

All animal operations were approved by the Animal Ethics Committee of the Plastic and Surgery Hospital of Peking Union University. Pregnant C57BL/6J mice at embryonic day 10.5 (35–39 somites), 11.5 (45–47 somites), and 12.5 (48–51 somites) were sacrificed, respectively, and embryonic lambdoidal junction tissues obtained as previously described (Li et al., 2019). For each time point, lambdoidal junction tissues from 17 or 18 embryos were pooled, with three replicates (17–18 embryos pooled per replicate). The samples were ground in liquid nitrogen, extracted using TRIzol reagent (Invitrogen), and purified using an RNeasy kit (Qiagen). An RNA Nano 6000 Assay Kit (Agilent Technologies, CA, United States) was used for RNA quantification and qualification. The RNASeq libraries were constructed following the manufacturer’s instructions. Illumina TruSeq RNA Sample Prep Kit (Cat#FC-122-1001) was used with 1 ug of total RNA to construct sequencing libraries. The cDNA library fragments were purified using the AMPure XP system (Beckman Coulter, Beverly, United States). A Qubit2.0 Fluorometer and qRT-PCR were used for the library qualification and quantification, respectively. RNA sequencing was performed on an Illumina NovaSeq 6000 platform.

### 2.2 Data analysis

Sequenced reads were first subjected to quality check using FASTQC (.19.7) (Brown et al., 2017) and then mapped to the reference genome (GRCm38/mm10) using Hisat2 (v2.0.5). Reads numbers were counted using the FeatureCounts (v1.5.0-p3). The FPKM of each gene was calculated based on the length of the gene and read counts mapped to the gene.

### 2.3 Differential expression analysis

Differential expression analysis of each set of groups was performed using the DESeq2 R package (1.20.0) (Love et al., 2014) using cutoffs of padj<=.05 and |log2 (foldchange)| ≥ 1. Gene Ontology (GO) and Kyoto Encyclopedia of Genes and Genomes (KEGG) enrichment analysis of differentially expressed genes was implemented using the clusterProfiler R package (3.8.1) (Yu et al., 2012). padj ≤ .05 was considered statistically significant.

### 2.4 Pathway analysis

For Gene Set Enrichment Analysis (GSEA) based on KEGG analysis using the clusterProfiler R package, the genes were first ranked before testing the selected gene sets. padj ≤ .05 was considered statistically significant.

### 2.5 Alternative splicing (AS) and protein-protein interaction (PPI) analysis

We used rMATS (4.1.0) software (Shen et al., 2014) to analyze AS events. The PPI network of DEGs was predicted using the Search Tool for the Retrieval of Interacting Genes (STRING) database. An interaction score threshold of .4 was set as the cutoff criterion. The PPI network was mapped using Cytoscape (version 3.7.1).

### 2.6 Quantitative real-time PCR (qPCR) and reverse transcription PCR (RT-PCR)

Total RNA was obtained using a TRIzol reagent (Invitrogen), followed by reverse transcription using the PrimeScript^®^ RT reagent kit (TaKaRa). Then, qRT-PCR was performed on a LightCycler^®^480 instrument (Roche, Swiss) and a SYBR Premix ExTaqII (TliRNaseHPlus) kit (Takara). The experimental data were processed by the 2^−ΔΔCT^ method. The expression of relative genes was normalized to β-actin. All primers are listed in Supplementary Table S1 (qPCR primers). For reverse transcription PCR, the above cDNA was further amplified with the specific primers listed in Supplementary Table S1 (RT-PCR primers). The RT-PCR products were run in 1%–5% agarose gels.

### 2.7 Immunostaining

Immunostaining was performed as described previously (Liu et al., 2019b). After antigen retrieval and blocking with serum for 1 h, the slices were incubated with the primary antibodies at 4°C overnight; these antibodies included anti-inhba (Santa Cruz, sc-166503), anti-wnt3a (Santa Cruz, sc-74537), anti-col6a1 (Santa Cruz, sc-377143), anti-wnt16 (Santa Cruz, sc-271897), anti-fgf18 (Santa Cruz, sc-393471), anti-chrm1 (Santa Cruz, sc-365966), anti-ngf (Abcam, ab52987), and anti-gdf7 (Abcam, ab189928). After washing three times with PBS, the slices were subjected to related secondary antibodies for 1 hour at room temperature. They were then washed three times with PBS (5 min each time), followed by incubation with diamidino-phenyl-indole (DAPI) for 30 min. Images were taken by laser scanning confocal fluorescence microscopy (Leica SP8).

### 2.8 RNAScope analysis

RNAScope assays [Advanced Cell Diagnostics (ACD) Biosystems] were performed to localize and detect the expression levels of target genes in lambdoidal junction tissues. The assays were performed according to the manufacturer’s instructions. Lambdoidal junction tissues were acquired and immersed in 4% paraformaldehyde solution at 4°C for 24 h. Then, the tissues were subjected to sterile PBS containing 10%, 20%, and 30% sucrose until they sank to the bottom. OCT-embedded sections (7 μm) were cut, adhered to SUPERFROST^®^ Plus chamber slides, and stored at −80°C. After washing with PBS for 5 min, the slides were incubated in hydrogen peroxide at 37°C for 10 min. After completely rinsing with distilled water, the slides were incubated with RNAScope 1 × Target Retrieval Reagent for 5 min at 100°C, followed by washing with distilled water and incubation in 100% alcohol for 3 min. The slides were then completely dried at room temperature. An ImmEdge hydrophobic barrier pen was used to draw a circle around each slice. The dry slides were then placed onto an EZ-Batch Slide Holder and five drops of Protease III were added to completely cover the tissues. After incubating with Protease III at 40°C for 30 min, the slides were rinsed with distilled water. Four drops of probe mix were then added to each section and incubated for 2 h at 40°C. The four targeting probes were *Krt5* (GenBank accession number NM_027011.2, Cat No. 415041, ACD) in channel C1, *Eno3* (GenBank accession number NM_007933.3, Cat No. 809781-C2, ACD) and *Lgals1* (GenBank accession number NM_008495.2, Cat No. 897151-C2, ACD) in channel C2, and *Col1a1* (GenBank accession number NM_007742.3, Cat No. 319371-C3, ACD) in channel C3. The slides were washed for 2 min with washing buffer at room temperature, then sequentially subjected to five drops of RNAScope Multiplex AMP 1 (30 min at 40°C), AMP 2 (30 min at 40°C), and AMP3 (15 min at 40°C). We used a nucleic acid fluorescent dye (Opal 520, Cat No. Akoya.A520, ACD) to mark channels C1, C2, and C3. The slides were then washed in 1× washing buffer twice between incubations, and then incubated with three to five drops of DAPI for 30 s at room temperature. Fluorescent images were acquired by laser scanning confocal fluorescence microscopy.

### 2.9 Statistical analysis

Statistical analysis was performed using GraphPad Prism 8.0 software. Student’s t-test (unpaired, two-tailed) was used to analyze the statistical differences between the two comparison groups. *p* < .05 was considered statistically significant.

## 3 Results

### 3.1 Differential expression during upper lip and primary palate development

**FIGURE 1:**
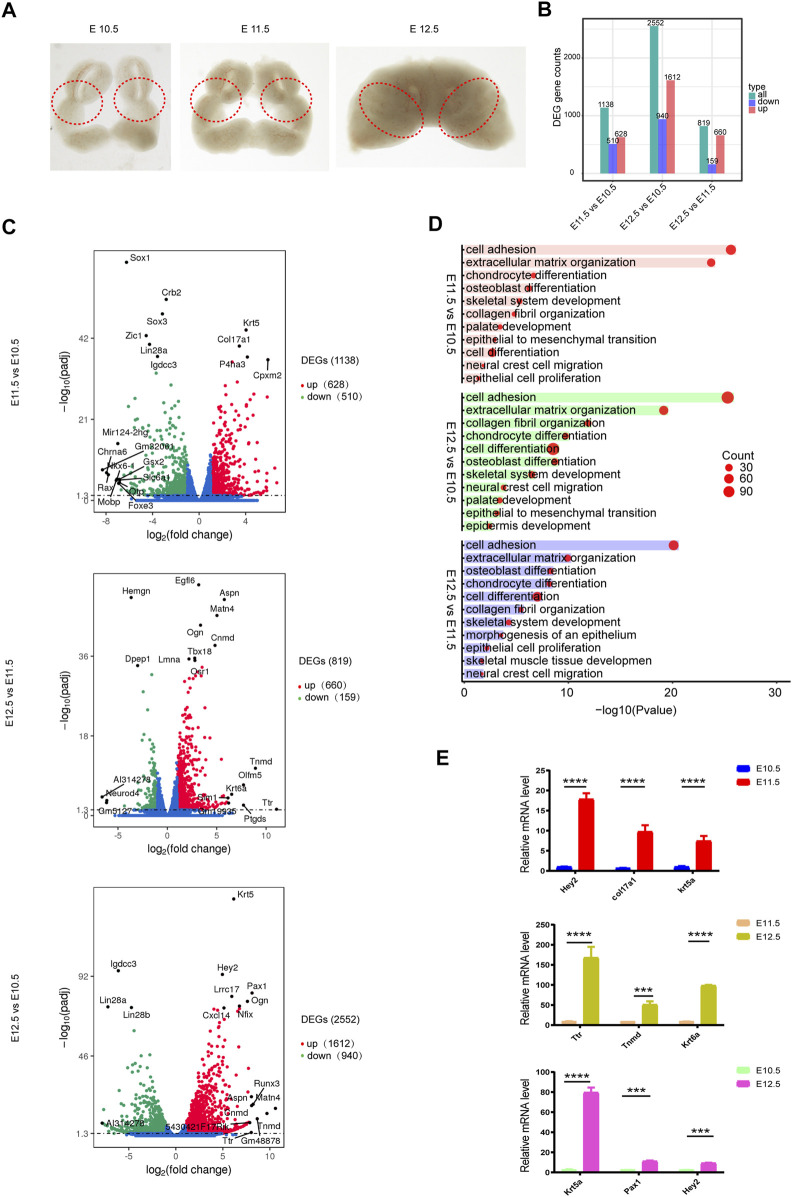
Transcriptomic changes during upper lip and primary palate development. **(A)** Lambdoidal junction tissues of mice at embryonic days 10.5, 11.5, and 12.5. **(B)** Histogram showing upregulated and downregulated DEGs for comparison groups E11.5 vs. E10.5, E12.5 vs. E10.5, and E12.5 vs. E11.5, respectively. **(C)** Volcano plots showing upregulated and downregulated DEGs in comparison group E11.5 vs. E10.5 (upper), E12.5 vs. E11.5 (middle), and E12.5 vs. E10.5 (lower), respectively. **(D)** Gene ontology analysis of upregulated DEGs in comparison group E11.5 vs. E10.5 (upper), E12.5 vs. E10.5 (middle), and E12.5 vs. E11.5 (lower), respectively. Circle size: gene number; bar length: range of *p* values. **(E)** Quantitative PCR validation of representative genes. *p < .05.

To investigate transcriptomic changes during the fusion of the upper lip and primary palate (Figure 1A), we calculated the FPKM expression values for differentially expressed genes (DEGs) and compared their fold-change values among different time points. FDR ≤ .05 and |log2 (foldchange)| ≥ 1 were set as the threshold values. Differential expression analysis identified 1138, 2552, and 819 DEGs in E11.5 vs. E10.5, E12.5 vs. E10.5, and E12.5 vs. E11.5, respectively. Further analysis of three comparison groups (E11.5 vs. E10.5, E12.5 vs. E10.5, and E12.5 vs. E11.5) identified 628, 1,612, and 660 upregulated genes and 510, 940, and 159 downregulated genes, respectively (Figure 1B, Supplementary Table S2).

In these three comparison groups, the upregulated DEGs were mainly associated with cell adhesion (*Col16a1*, *Col12a1*, etc.) (Chen et al., 2014), extracellular matrix organization (*Col17a1*, *Col16a1*, etc.) (Grässel and Bauer, 2013), chondrocyte differentiation (*Osr2*, *Wnt10b*, etc.) (Lam et al., 2013), cell differentiation (*Foxa1*, *Sema5a*, etc.) (Katoh et al., 2013), skeletal system development (*Tgfb2, Fst*, etc.) (Twine et al., 2014), palate development (*Pdgfra*, *Wnt11*, etc.) (Lee et al., 2008), etc (Figures 1C,D, Supplementary Table S3). Khan et al. (2019) reported a smaller collagen fibril diameter and a higher collagen number density and fibril-area fraction on the medial side of cleft lip tissue, indicating the potential role of collagen in CL/P. We then performed qPCR validation of the top candidate upregulated DEGs (*Krt5*, *Pax1*, *Hey2*, *Ttr*, *Krt6a*, and *Tnmd*) involved in organic development between different comparison groups (Figure 1E).

### 3.2 Validation of development-associated DEGs by RNAScope

**FIGURE 2:**
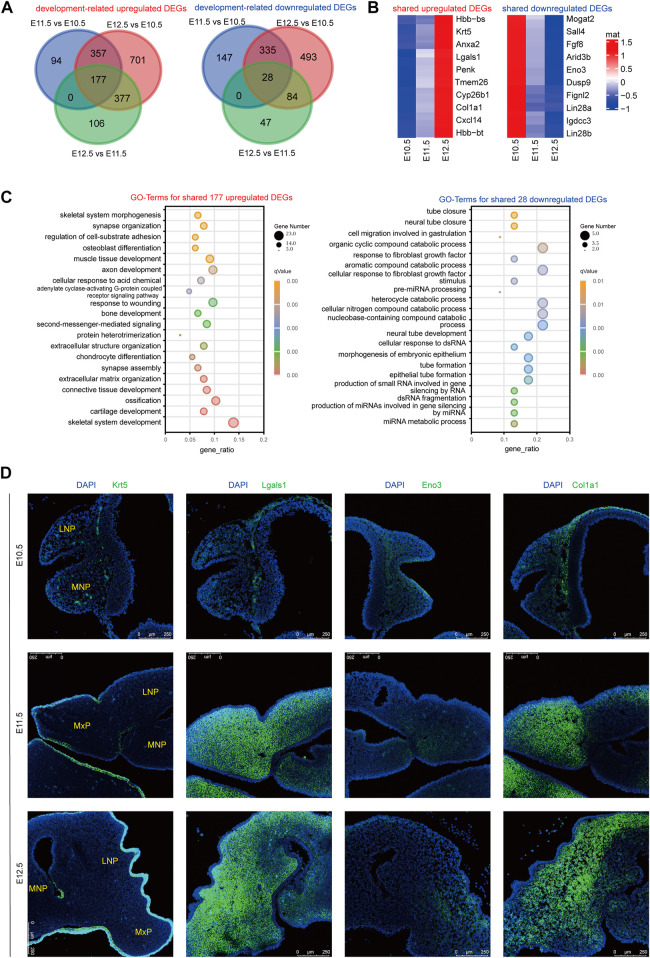
RNAScope validation of development-associated upregulated or downregulated DEGs. **(A)** Venn diagram showing the amount of shared upregulated or downregulated DEGs among comparison group E11.5 vs. E10.5, E12.5 vs. E11.5, and E12.5 vs. E10.5. **(B)** Heatmap showing the top ten shared upregulated or downregulated DEGs among E10.5, E11.5, and E12.5. Color key: expression levels, **(C)** GO terms for shared upregulated (left) and downregulated (right) DEGs from E10.5 to E12.5. **(D)** RNAScope analysis of the location and expression levels of candidate genes among E10.5, E11.5, and E12.5. MxP: maxillary prominence; LNP: lateral nasal; MNP: medial nasal.

We further profiled the consistently upregulated and downregulated DEGs from E10.5 to E12.5 (Figure 2A, Supplementary Table S4). Among the top 10 upregulated DEGs (Figure 2B), *Lgals1* reportedly regulates cell growth, migration, and proliferation (Wu et al., 2019); *Col1a1* is involved in collagen formation; and *Krt5* facilitates epithelial tissue differentiation. The persistently downregulated gene *Eno3* plays an important role in muscle development and regeneration (Merkulova et al., 1997). The 177 shared upregulated genes were mainly correlated with skeletal system development, ossification, connective tissue development, and extracellular matrix organization (Figure 2C). 28 The shared downregulated genes were mainly related to neural tube closure. To validate the bioinformatic analysis of the expression profiles and confirm the exact location of these DEGs in lambdoidal junction tissues, RNAScope was used to profile the location and expression levels of *Lgals1*, *Col1a1*, *Krt5*, and *Eno3* (Figure 2D). As expected, the results basically matched those of our bioinformatic analysis. *KRT5*, a known periderm marker, was mainly expressed in the superficial layer of the epidermis. *Col1a1*, important for fibril-forming collagen production, was mainly expressed in the mesenchyme (Figure 2D). The results revealed that our RNA sequencing data reliably recognized potential differentially expressed genes in upper lip and primary palate development.

### 3.3 Important signaling pathways and relative DEGs involved in upper lip and primary palate development

**FIGURE 3:**
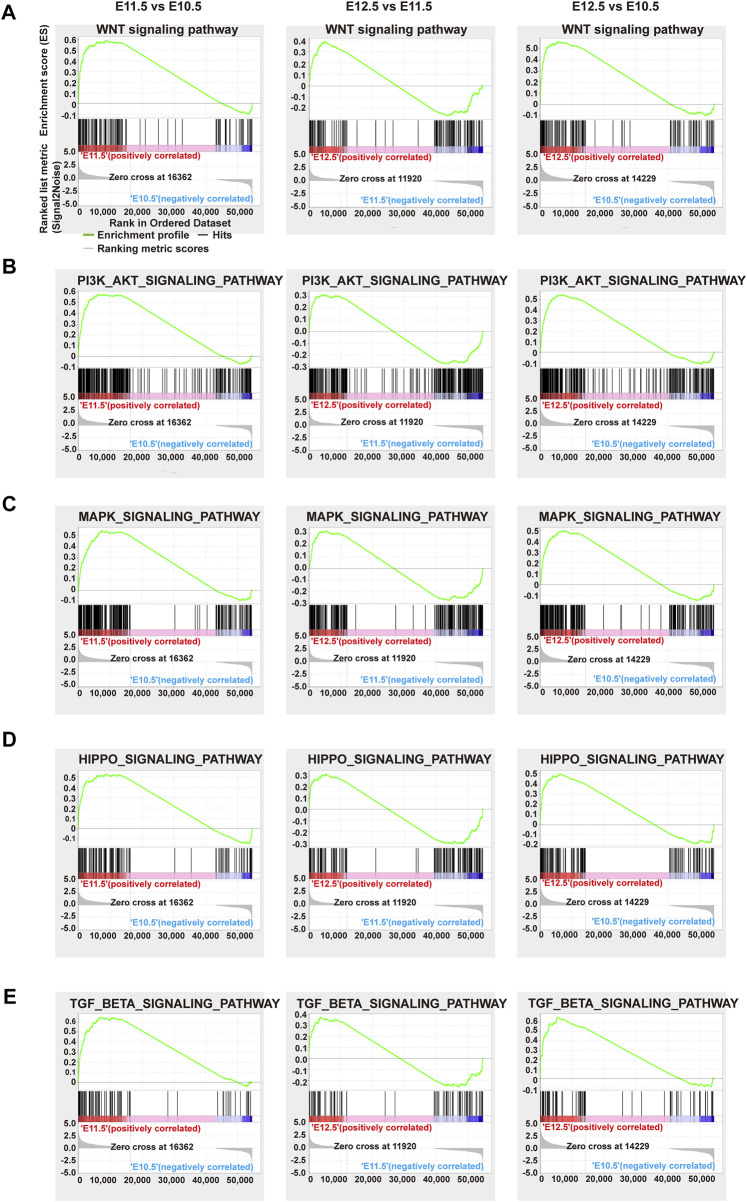
GSEA analysis of representative significant signal pathways. Gene set enrichment plots of the **(A)** Wnt, **(B)** PI3K-AKT, **(C)** MAPK, **(D)** Hippo, and **(E)** Tgf-beta signaling pathways among comparison groups E11.5 vs. E10.5 (left), E12.5 vs. E11.5 (middle), and E12.5 vs. E10.5 (right), respectively. Green curve: enrichment score; vertical red or blue bars: gene positions.

We further performed gene set enrichment analysis (GSEA) on different comparison groups based on KEGG analysis (Supplementary Table S5). As shown in Figure 3, from E10.5 to E12.5, the genes correlated with the Wnt, PI3K-Akt, MAPK, Hippo, and TGF-beta signaling pathway showed a trend toward up-regulation. Several Wnt signaling-related genes are reportedly involved strongly in orofacial clefts (Reynolds et al., 2019). Excessive intake of retinoic acid (RA) could cause cleft palate and PI3K-Akt signaling was related to the action of RA, implying the possible influence of PI3K-Akt signaling on palate development (Hu et al., 2013). MAPK signaling affected palate development in animal studies (Liu et al., 2019a). Differences in DNA methylation have been reported in many candidate genes contributing to non-syndromic cleft upper lip with or without cleft palate (NSCL/P), meanwhile, common differential methylation was also found in genes correlated with the Hippo signaling pathway, indicating an association between this pathway and NSCL/P (Young et al., 2021). The TGF-beta signaling pathway is essential for the morphogenesis of craniofacial tissues, the failure of which can cause severe craniofacial defects (Dudas et al., 2006).

**FIGURE 4:**
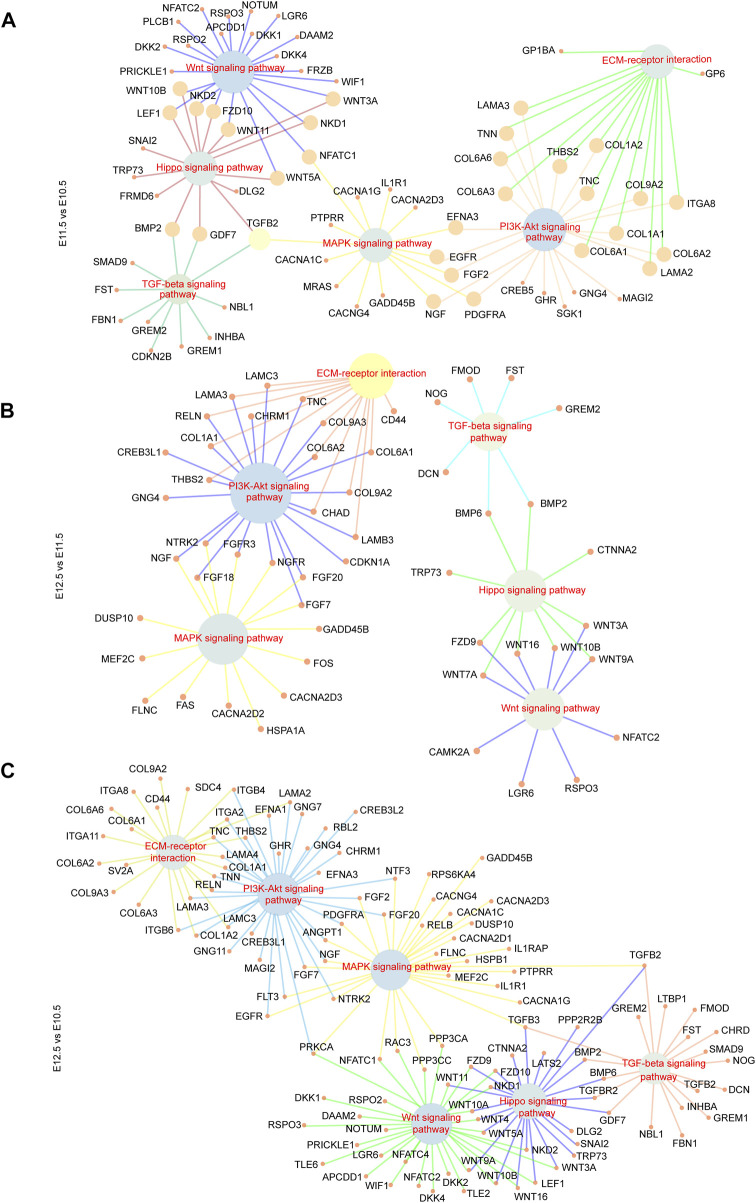
Signal-gene interaction network. DEGs and significant signal pathways playing essential roles during upper lip and primary palate development among comparison groups **(A)** E11.5 vs. E10.5, **(B)** E12.5 vs. E11.5, and **(C)** E12.5 vs. E10.5 were chosen, and profiled by constructing signal-gene networks, respectively. Inner nodes: signal pathways; surrounding nodes: genes; lines between inner and surrounding nodes: interactions between signaling pathways and genes.

**FIGURE 5:**
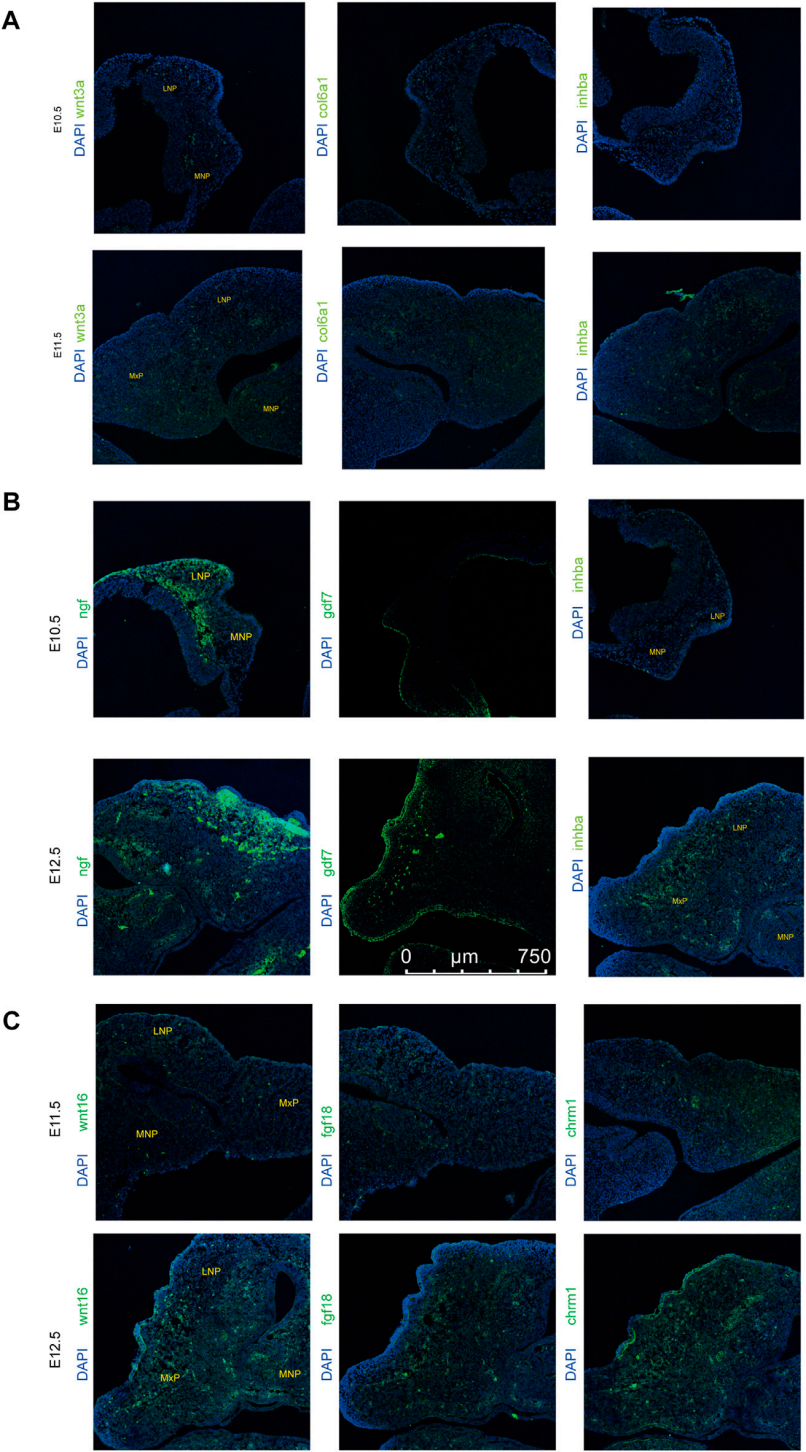
xpression levels of representative genes from the signal-gene network detected by immunofluorescence in lambdoidal junction tissues of mice at embryonic days 10.5, 11.5, and 12.5. **(A)** Expression levels of wnt3a, col6a1, and inhba between E10.5 and E11.5. **(B)** Expression levels of wnt16, fgf18, and chrm1 between E12.5 and E11.5. **(C)** Expression levels of gdf7, inhba, and ngf between E12.5 and E10.5. MxP: maxillary prominence; LNP: lateral nasal; MNP, medial nasal.

To further investigate the relationship between the above signaling pathways and related DEGs that might play important roles during lip and primary palate development, we constructed a signal-gene network based on KEGG analysis (Figure 4). We selected 138 significant DEGs involving the above five pathways as potential targets during upper lip and primary palate development (Supplementary Table S6). Among these DEGs, *Tnn, Gdf7, Inhba, Col6a6, Cdkn2b, Wnt3a, Wnt10b, Tgfb2*, *Col6a1*, and *Col1a1* in comparison groups E11.5 vs. E10.5*; Wnt16, Dcn, Fgf18, Chrm1, Lama3, Bmp6, Tnc, Gng4, Chad*, and *Col9a3* in comparison group E12.5 vs. E11.5*;* and *Gdf7, Tnn, Flt3, Inhba, Gng4*, *Ngf*, *Lama3, Col6a1, Cacna2d3*, and *Tnc* in comparison group E12.5 vs. E10.5 showed relatively high fold-changes in expression levels. Immunofluorescence analysis to explore the location and relative expression levels during upper lip and primary development (Figure 5) revealed Wnt3a and Col6a1 expression in the mesenchyme, Ngf expression mainly in the lateral nasal prominence, and Gdf 7 expression mainly in the superficial layer of the epidermis (Figure 5).

### 3.4 Alternative splicing events during upper lip and primary palate development

**FIGURE 6:**
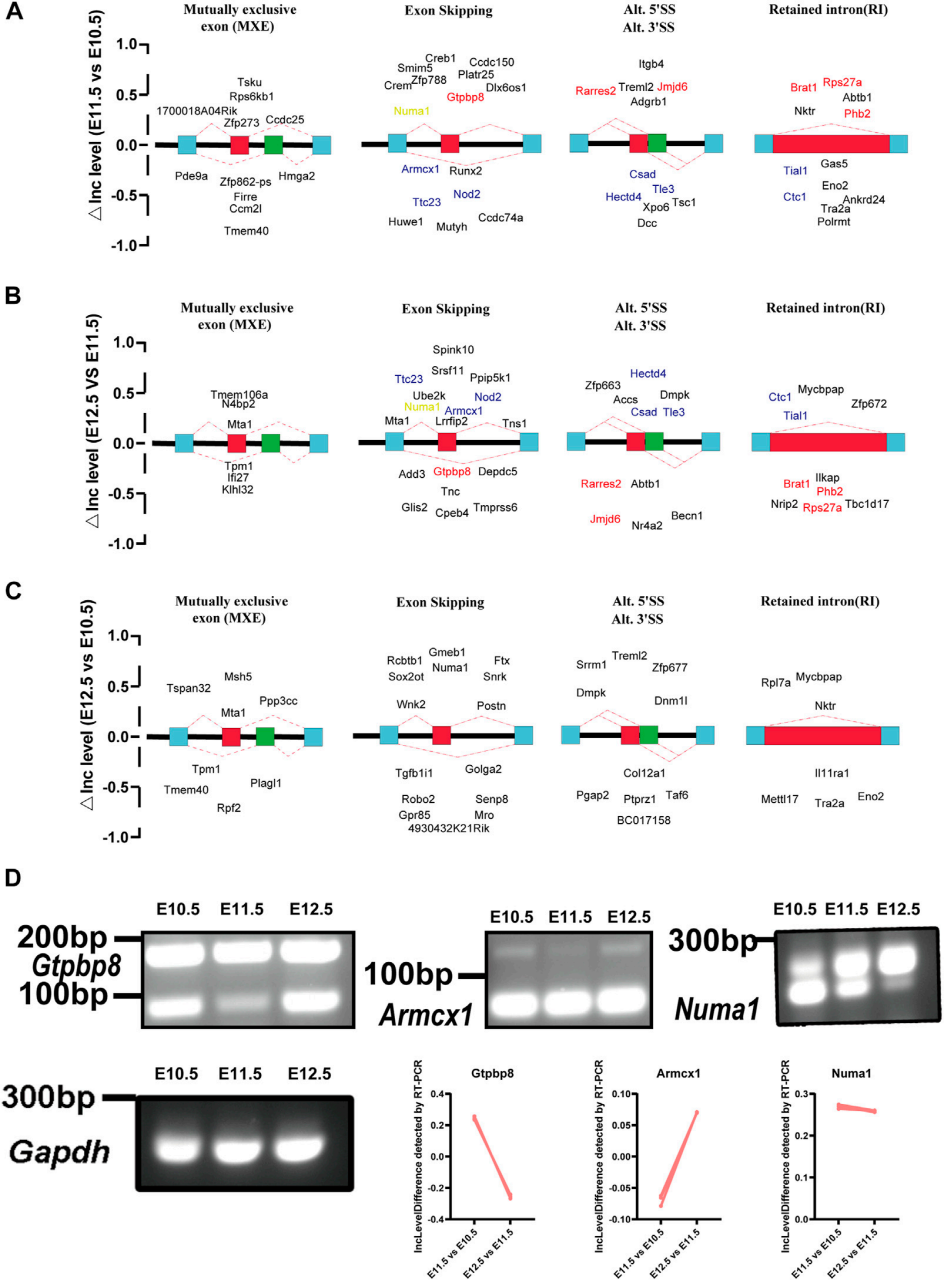
Changes in alternative splicing events in lambdoidal junction tissues of mice at embryonic days 10.5, 11.5, and 12.5. Development-associated changes in alternative splicing events among comparison groups **(A)** E11.5 vs. E10.5, **(B)** E12.5 vs. E11.5, and **(C)** E12.5 vs. E10.5. Red: changes of △Inc level that first increased and then decreased; blue: △Inc level first decreased and then increased; orange: △Inc level of Numa1 persistently upregulated from E10.5 to E12.5. **(D)** RT-PCR validation to demonstrate the expression levels of *Numa1* from E10.5 to E12.5. IncLevel Difference (△IncLevel) = IncLevel1 − IncLevel2; IncLevel1 = the expression of exon inclusion isoform/the expression of two isoforms (in experimental group 1), IncLevel2 = expression of exon inclusion isoform/expression of two isoforms (in experimental group 2).

**FIGURE 7:**
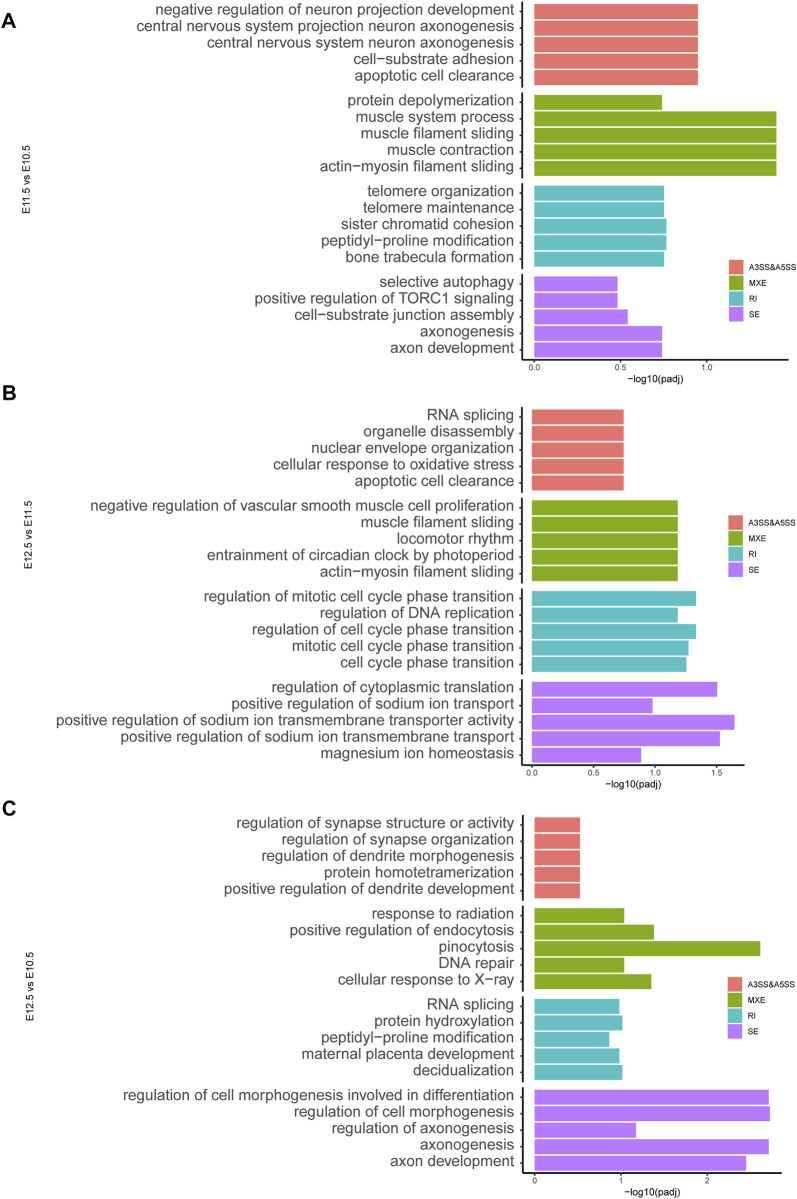
ene ontology analysis showing the top five ranked terms for alternatively spliced genes among comparison groups **(A)** E11.5 vs. E10.5, **(B)** E12.5 vs. E11.5, and **(C)** E12.5 vs. E10.5.

To investigate molecular features of upper lip and primary palate development from E10.5 to E12.5, we analyzed potential alternative splicing events (Figure 6). We identified 210, 329, and 131 AS events for E11.5 vs. E10.5, E12.5 vs. E10.5, and E12.5 vs. E11.5, respectively. These AS events include alternative 3′ splice sites (A3′SSs), alternative 5′ splice sites (A5′SSs), mutually exclusive exons (MXEs), retained introns (RIs), and skipped exons (SEs) (Figures 6A–C and Supplementary Table S7). SEs and MXEs were the primary AS events. In E11.5 vs. E10.5, the upregulated AS genes were mainly associated with cell adhesion, muscle system processes, bone trabecula formation, and axon development. In E12.5 vs. E11.5, the upregulated AS genes were strongly involved in organelle disassembly, muscle filament sliding, regulation of cell cycle phase transition, and regulation of cytoplasmic translation. In E12.5 vs. E10.5, the upregulated AS genes were primarily correlated with pinocytosis, regulation of cell morphogenesis involved in differentiation, and axonogenesis. We also conducted RT-PCR to demonstrate candidate AS changes (Figure 6D). Gene Ontology (GO) analysis of AS genes showed distinctly enriched ontologies (Figure 7). Overall, we uncovered previously unrecognized AS signatures during upper lip and primary palate development and constructed a reference dataset for cleft lip and palate.

### 3.5 Protein-protein interaction network (PPI) analysis of development-associated DEGs

**FIGURE 8:**
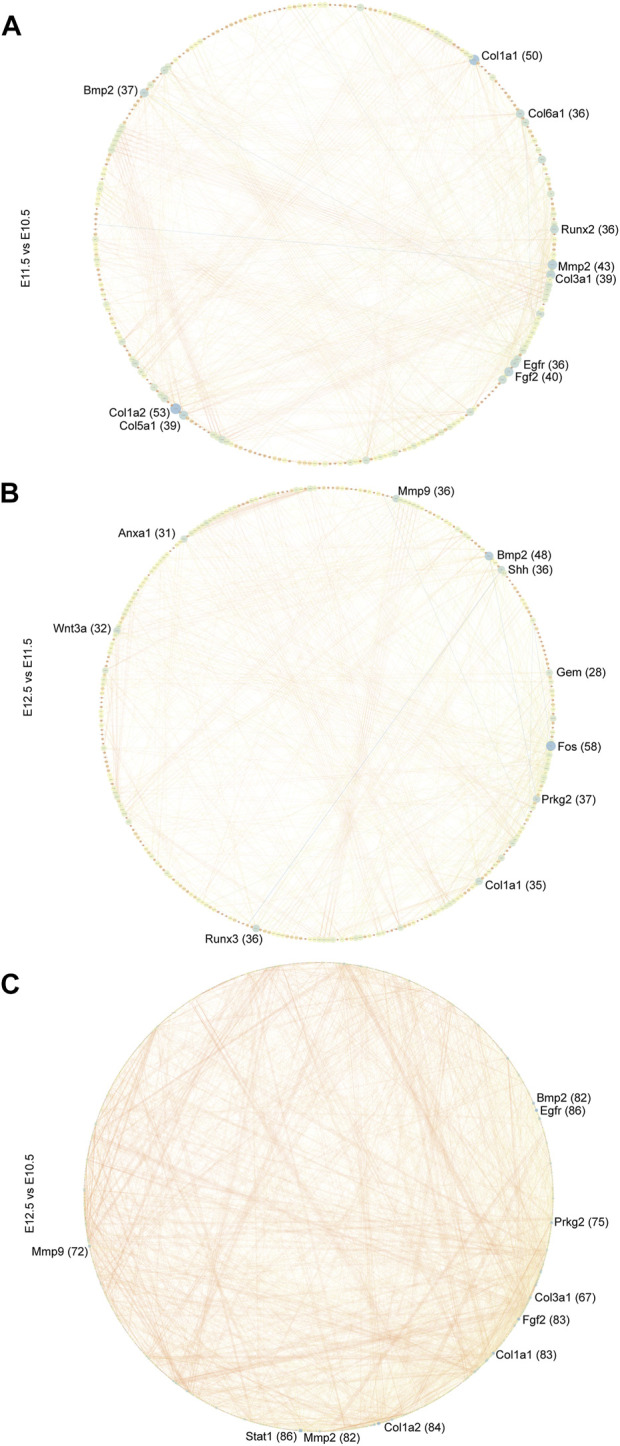
PPI network of developmentally related DEGs. PPI network of DEGs among comparison groups **(A)** E11.5 vs. E10.5, **(B)** E12.5 vs. E11.5, and **(C)** E12.5 vs. E10.5 constructed in Cytoscape. The top 10 hub nodes (genes) are marked. The numbers in parentheses next to the genes indicate the node degrees.

**FIGURE 9:**
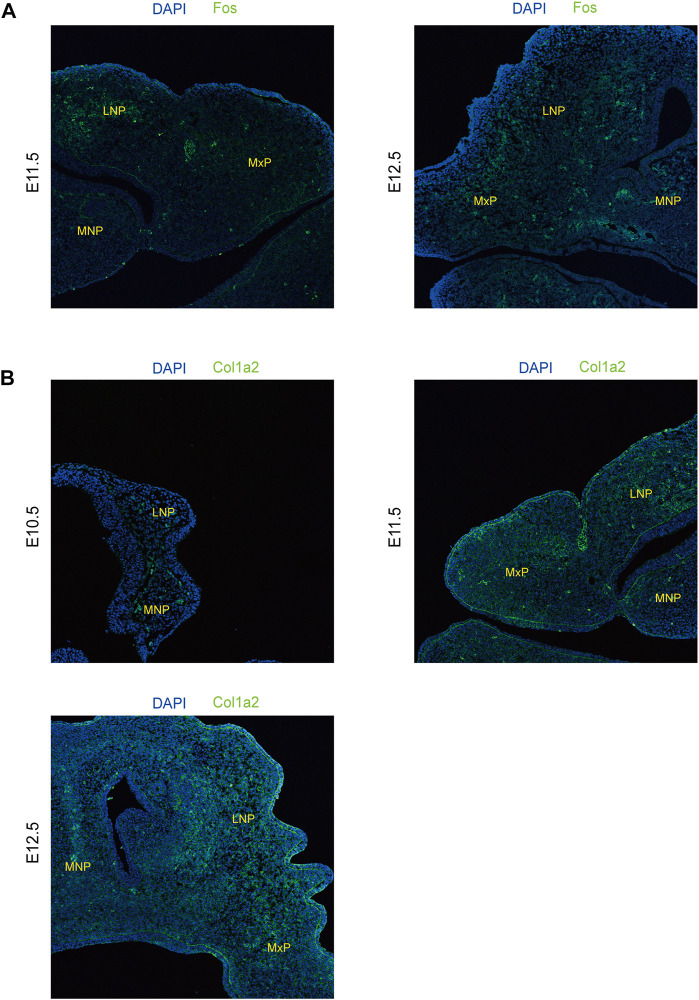
xpression levels of representative genes from the PPI network detected by immunofluorescence in lambdoidal junction tissues of mice at embryonic days 10.5, 11.5, and 12.5. **(A)** Expression levels of col1a2 at E10.5, E11.5, and E12.5. **(B)** Expression levels of Fos between E11.5 and E12.5. MxP: maxillary prominence; LNP: lateral nasal; MNP: medial nasal.

To further explore the biological functions of development-associated upregulated DEGs, we performed PPI network analyses of DEGs based on the STRING database. DEGs filtered using the threshold values (FDR ≤ .05, |Log2FC| ≥ 2) revealed 1,476 interaction relationships among 399 genes among the DEGs from comparison group E11.5 vs. E10.5 (Figure 8A). The DEGs *Col1a2*, *Col1a1, Mmp2, Fgf2, Col3a1, Col5a1, Bmp2, Egfr, Runx2*, and *Col6a1* played core roles in the whole network. The analysis additionally identified 10,089 interaction relationships within 443 genes within the DEGs from comparison group E12.5 vs. E11.5 (Figure 8B). The DEGs *Fos, Bmp2, Prkg2, Runx3, Mmp9, Shh, Col1a1, Wnt3a, Anxa1*, and *Gem* played key roles in the whole network. Furthermore, we identified 6433 interaction relationships among 1,176 genes in DEGs from comparison group E12.5 vs. E10.5 (Figure 8C). The DEGs *Stat1, Egfr, Col1a2, Fgf2, Col1a1, Mmp2, Prkg2, Bmp2, Mmp9*, and *Col3a1* played essential roles in the whole network. The degrees of network centrality of the genes are presented in Supplementary Table S8. The DEGs Wnt3a, Shh, and Bmp2 affect the development of the upper lip and palate; mutations in these DEGs could induce the CL/P (Dixon et al., 2011). Immunofluorescence analysis was performed to explore the location and relative expression levels of hub genes like Fos and col1a2 (Figure 9). Col1a2 was mainly expressed in the mesenchyme (Figure 9). Our results provided a range of potential target genes involved in the development of the upper lip and primary palate. Alterations of these genes might lead to CL/P.

## 4 Discussion

CL/P is one of the most frequent birth defects. Two main etiological factors of CL/P are genetic and environmental (Dixon et al., 2011). Similarly, two major approaches are used to explore congenital disorders, namely, genetics and developmental approaches (Li et al., 2020). Regarding genetics approaches, most monogenetic congenital diseases are investigated by identifying series of pathogenic genes and their mutations in development. However, polygenic diseases like CL/P, involve many etiological factors. Thus, the exploration of CL/P pathogenesis through development approaches is important, and the in-depth study of the normal developmental processes of upper lip and primary development is essential.

As E10.5–11.5 was the early fusion period of the upper lip and primary palate, it is a critical period in upper lip and primary palate development. Therefore, we detected the largest number of DEGs at this stage, most of which were involved in cellular structures such as cell adhesion, extracellular matrix organization, and collagen fibril organization. At this stage, some DEGs associated with neural crest cell migration, including *Sema5a*, *Zeb2*, *Nrp2*, and *Pax3*, were upregulated and chondrocyte and osteoblast differentiation may have occurred (Figure 1D). Moreover, epithelial fusion and the epithelial-to-mesenchymal transition occurred among the MNP, MxP, and LNP(Losa et al., 2018). In our results, the DEGs correlated with epithelial cell proliferation (*Ehf*, *Wnt5a*, *Col8a2*, *Col8a1*, *Fgf2*, *Egfr*, and *Runx2*) and the epithelial-to-mesenchymal transition (*Tgfb2*, *Bmp2*, *Wnt11*, *Lef1*, *Snai2*, *Nfatc1*, and *Msx1*) were upregulated from E10.5 to E11.5 (Supplementary Table S3). By E12.5, the fusion processes are essentially complete, and skeletal systems, muscles, and other soft tissues emerge (Li et al., 2019). Our results showed that DEGs involved in cellular structures, morphogenesis of the epithelium, and skeletal system development were continuously upregulated from E11.5 to E12.5, indicating the persistent tissue fusion and formation of the skeletal system (Figure 2C, left). In addition, as neural tube closure of mice begins at E8.5 and is almost complete at E9.5, our results showed a downregulation of genes related to neural tube closure (*Trim71*, *Cecr2*, and *Sall4*) (Figure 2C, right). The location and validation of the expression levels of candidate genes by RNAScope further confirmed the credibility of our results (Figure 2D). Although the sequenced tissues were from the lambda region, the distribution of candidate genes was spread out over three prominences, indicating that these genes may be involved in events other than upper lip and palate fusion.

Signaling pathways play important roles in multi-organ development and congenital malformations. The Wnt, MAPK, Hippo, and TGF-beta signaling pathways are involved in the development of the lip and/or palate and CL/P (Zhu et al., 2012; Liu et al., 2019a; Reynolds et al., 2019). In our results, the PI3K-Akt and Hippo signaling pathways also intensified from E10.5 to E12.5. PI3K-Akt signaling was correlated with the action of RA, which causes cleft palate, signifying the association between PI3K-Akt signaling and palate development (Hu et al., 2013). Genes correlated with the Hippo signaling pathway were commonly methylated, and differences in gene methylation were strongly associated with NSCL/P, indicating the correlations between the Hippo signaling pathway and NSCL/P (Young et al., 2021). Further analysis of the signal-gene network revealed highly upregulated DEGs associated with these significant pathways, including *Tgfb2*, *Wnt16*, *Fgf18*, *Wnt3a*, *Col6a1*, and *Inhba*, providing a series of candidate genes for normal development or CL/P. Among these genes, *Tgfb2*, *Wnt16*, and *Inhba* are important for palatogenesis (Zhu et al., 2012; Liu et al., 2019a). *Fgf18* and *Wnt3a* were associated with non-syndromic cleft lip with or without palate (NSCL/P) (Riley et al., 2007; Reynolds et al., 2019). *Col6a1* is essential for skeletal muscle development (Cescon et al., 2018). *Chrm1* and *Ngf* are important for nervous system development (Renz et al., 2018; Magrì et al., 2021).

Through alternative splicing, a single gene can produce more than one mRNA transcript. During normal tissue and organic development, many AS events occur. Disorders in AS can lead to abnormalities in tissue functions and identity (Baralle and Giudice, 2017). A study on the mouse cerebral cortex development observed approximately 400 alternative splicing events in the adult cerebral cortex and embryos and reported that nearly one-third of genes that were differentially regulated by alternative splicing showed no alterations in total expression levels (Dillman et al., 2013). Studies on skeletal muscle development have reported that specific RNA-binding proteins like PTB, Qk, and RBFOX2 coordinate muscle-specific AS events (Hall et al., 2013; Singh et al., 2014). Particularly, RBFOX2 depletion caused myoblast fusion defects (Singh et al., 2014). We also identified a series of AS events during the upper lip and primary palate development and genes that may be involved in the upper lip and primary palate development, including *Gtpbp8*, *Jmjd6*, *Rarres2*, *Phb2, Armcx1, Ttc23, Nod2, Hectd4, Csad, Tle3*, and *Numa1*. These genes were largely associated with muscle system development, bone formation, and cellular differentiation, which was strongly correlated with the upper lip and primary palate development.

We further investigated the potential regulatory mechanisms during upper lip and primary palate development. DEGs PPI network analysis was conducted and the top 10 hub genes were identified, including *Col1a2, Col1a1, Mmp2, Fgf2, Col3a1, Col5a1, Bmp2, Egfr, Runx2*, and *Col6a1* in comparison group E11.5 vs. E10.5; *Fos, Bmp2, Prkg2, Runx3, Mmp9, Shh, Col1a1, Wnt3a, Anxa1*, and *Gem* in comparison group E12.5 vs. E11.5; *Stat1, Egfr, Col1a2, Fgf2, Col1a1, Mmp2, Prkg2, Bmp2, Mmp9*, and *Col3a1* in comparison group E12.5 vs. E10.5. Mutations in *Wnt3a, Shh*, and *Bmp2* reportedly induce CL/P (Dixon et al., 2011). Collagen-related genes like *Col6a1* and *Col1a2* are important for extracellular matrix organization. *Runx2* is an essential inducer of osteogenesis and chondrogenesis (Komori, 2018). Further in-depth investigation of these hub genes may identify novel predictive biomarkers and clinical therapeutic targets for the treatment of CL/P.

These data demonstrate that the development of the upper lip and primary palate is a complex and elaborate process. Our findings suggest that further research efforts might concentrate on these potential candidate genes in upper lip and primary palate development and CL/P pathogenesis.

## Data Availability

The data presented in the study are deposited in the GEO repository, accession number: GSE211601.

## Supplementary material

Supplementary Table S1 Primers for qPCR and RT-PCR validation.

Supplementary Table S2 DEGs for comparison groups E11.5 vs. E10.5, E12.5 vs. E11.5, and E12.5 vs. E10.5.

Supplementary Table S3 GO terms of DEGs for comparison groups E11.5 vs. E10.5, E12.5 vs. E11.5, and E12.5 vs. E10.5.

Supplementary Table S4 Upregulated and downregulated DEGs for Venn diagrams.

Supplementary Table S5 KEGG terms used for GSEA analysis.

Supplementary Table S6 Signal pathways and relative genes used for the construction of signal-gene network.

Supplementary Table S7 Detailed information on alternative splicing events.

Supplementary Table S8 Node centrality analysis of the PPI networks of groups E11.5 vs. E10.5, E12.5 vs. E11.5, and E12.5 vs. E10.5.

## Abbreviations

A3′SS, alternative 3′ splice site; A5′SS, alternative 5′ splice site; AS, alternative Splicing; CL/P, cleft upper lip/palate; DEGs, differentially-expressed genes; GO, gene Ontology; GSEA, gene set enrichment analysis; KEGG, Kyoto Encyclopedia of Genes and Genomes; LNP, the lateral nasal; MNP; the medial nasal; MxP, the maxillary prominence; MXE, mutually exclusive exon; NSCL/P, non-syndromic cleft upper lip with or without cleft palate; PPI, protein-protein interaction; qPCR, real-time quantitative polymerase chain reaction; RNA-Seq, RNA sequencing; RA, retinoic acid; RI, retained intron; RT-PCR, reverse transcription polymerase chain reaction; STRING, Search Tool for the Retrieval of Interacting Genes; SE, skipped exon.
